# Development and validation of a risk prediction model for work disability: multicohort study

**DOI:** 10.1038/s41598-017-13892-1

**Published:** 2017-10-19

**Authors:** Jaakko Airaksinen, Markus Jokela, Marianna Virtanen, Tuula Oksanen, Jaana Pentti, Jussi Vahtera, Markku Koskenvuo, Ichiro Kawachi, G. David Batty, Mika Kivimäki

**Affiliations:** 1 Finnish Institute of Occupational Health, Helsinki, Finland; 2 Department of Psychology and Logopedics, Faculty of Medicine, University of Helsinki, Helsinki, Finland; 3 Department of Public Health, University of Turku, Turku, Finland; 4 Clinicum, Faculty of Medicine, University of Helsinki, Helsinki, Finland; 5 Harvard T H Chan School of Public Health, Boston MA, USA; 6 Department of Epidemiology and Public Health, University College London, London, UK

## Abstract

Work disability affects quality of life, earnings, and opportunities to contribute to society. Work characteristics, lifestyle and sociodemographic factors have been associated with the risk of work disability, but few multifactorial algorithms exist to identify individuals at risk of future work disability. We developed and validated a parsimonious multifactorial score for the prediction of work disability using individual-level data from 65,775 public-sector employees (development cohort) and 13,527 employed adults from a general population sample (validation cohort), both linked to records of work disability. Candidate predictors for work disability included sociodemographic (3 items), health status and lifestyle (38 items), and work-related (43 items) variables. A parsimonious model, explaining > 99% of the variance of the full model, comprised 8 predictors: age, self-rated health, number of sickness absences in previous year, socioeconomic position, chronic illnesses, sleep problems, body mass index, and smoking. Discriminative ability of a score including these predictors was high: C-index 0.84 in the development and 0.83 in the validation cohort. The corresponding C-indices for a score constructed from work-related predictors (age, sex, socioeconomic position, job strain) were 0.79 and 0.78, respectively. It is possible to identify reliably individuals at high risk of work disability by using a rapidly-administered prediction score.

## Introduction

According to the International Labour Organization, over a billion people worldwide suffer from a disability and 80% of these persons are of working age^1^. In OECD countries, 6% of working-age people have exited the labor market due to a disability^2^. Apart from significant impacts on a person’s life chances, work disability is a problem for the society due to lost productivity. The prevention of work disability is becoming increasingly important due to population aging, as an increasing number of people are spending more years with functional health loss and disability, leading to an absolute expansion of morbidity^3^.

Recent studies of risk factors for work disability have confirmed the importance of older age^4^, low socioeconomic status^5,6^, a history of sickness absences^7,8^, unhealthy behaviors (smoking and high alcohol consumption)^9–11^, and stressful characteristics of work, such as high job strain and excessive job demands^12,13^. While these risk factors are useful in evaluating the risk of work disability at a population level, more sensitive and specific prediction instruments are needed to assess an individual’s disability risk. Multifactorial prediction algorithms or scores that take into account the combined effect of multiple risk factors have been used to identify people at high risk of chronic diseases, particularly cardiovascular disease^14–16^, but to date few multifactorial scores are available to identify those at increased risk of work disability. Multifactorial prediction models have been introduced for prediction of work disability in the US Army^17^ and sickness absence in working populations^18^. However, we are not aware of any validated multifactorial prediction score for work disability in the general working population. Such scores could facilitate the identification of individuals who are most likely to benefit from targeted interventions.

Using individual-level data on sociodemographic variables, health status and lifestyle, and work-related variables, we developed and validated two parsimonious and rapidly administered prediction algorithms for all-cause work disability, one based on all available data and the other using work-related variables only.

## Results

### Descriptive characteristics

**Figure 1:**
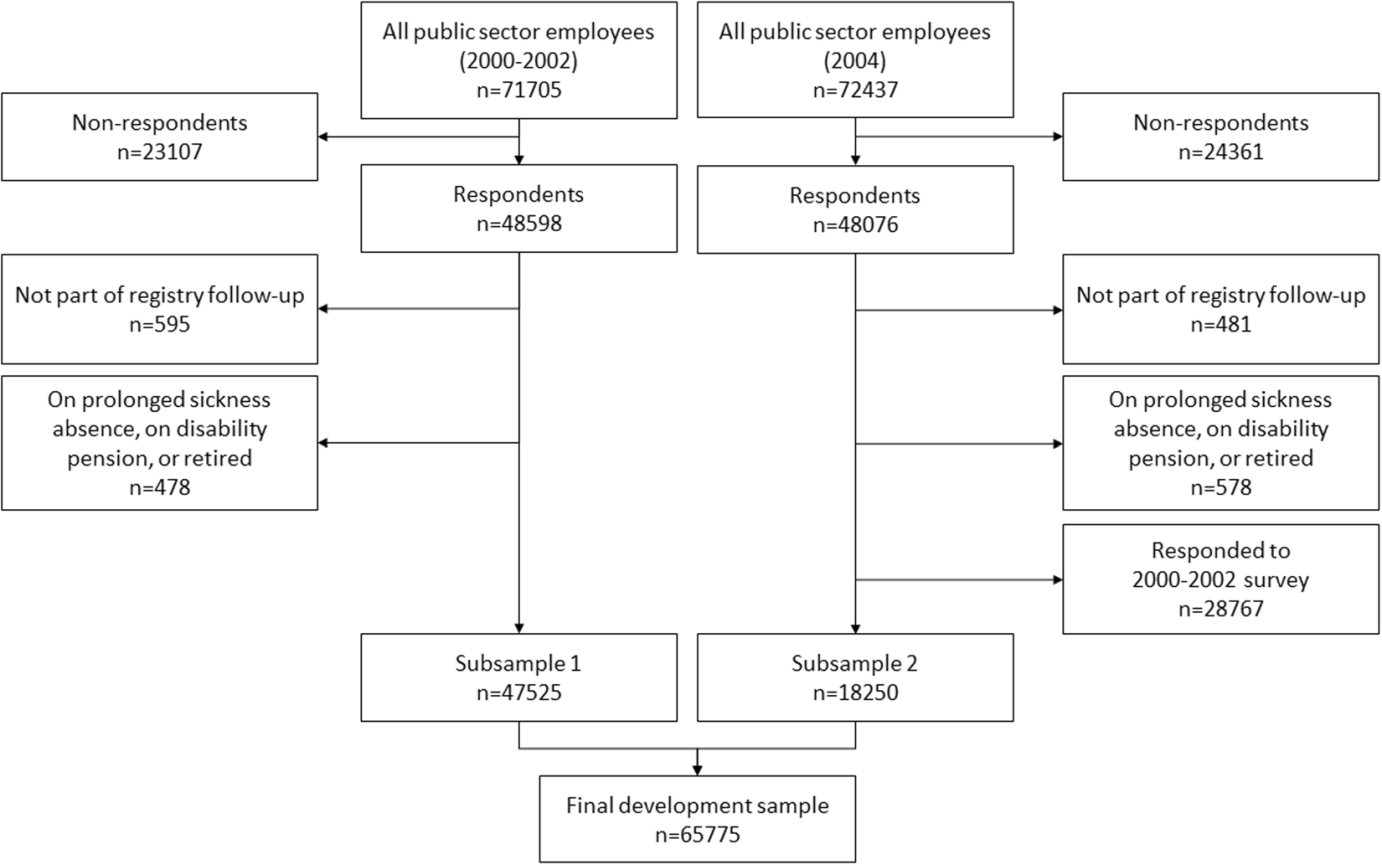
Sample selection flowchart for the development cohort.

We used data from two large prospective cohort studies, the Finnish Public Sector study (FPS) and the Health and Social Support (HeSSup) study^19–21^. Sample selection and the descriptive characteristics for the development (N = 65,775, mean age 43.7 years, 80% women) and validation (N = 13,527, mean age 39.5 years, 57% women) cohorts are provided in Fig. 1 and Table 1 (see Appendix 1 for the wordings of the questionnaire items). Overall, the development cohort and the validation cohort were quite similar, with the exception of more equal gender distribution and younger participants in the validation cohort. During the mean follow-up of 8.6 years, 5332 (8%) people were granted a disability pension in the development cohort. In the validation cohort mean follow-up was 9.5 years and 877 (6%) participants were granted disability pension. ICD-10 based reasons for granted full disability pensions are shown in Table 2.

**Table 1:** Descriptive statistics of the cohorts.

Statistic		FPS(2000) n = 47,525			FPS(2004) n = 18,250			FPS(2000 + 2004) n = 65,775			Hessup (1998) n = 13,527		
		Mean (SD)	No.	%	Mean (SD)	No.	%	Mean (SD)	No.	%	Mean (SD)	No.	%
Female			38413	81		37790	78		52599	80		7698	57
Age		44.57 (9.42)			41.41 (10.10)			43.70 (9.71)			39.52 (10.22)		
	< 35		8024	17		5117	28		13141	20		5496	41
	35–39		6161	13		2856	16		9017	14		0	0
	40–44		7948	17		2995	16		10943	17		4072	30
	45–49		8517	18		2758	15		11275	17		0	0
	50–54		9227	19		2327	13		11554	18		3959	29
	55+		7648	16		2197	12		9845	15		0	0
Socioeconomic position		3.77 (1.70)			3.70 (1.67)			3.75 (1.72)			3.72 (1.84)		
	1		1288	3		324	2		1612	2		2212	16
	2		12440	26		5060	28		17500	27		327	2
	3		12388	26		5391	30		17779	27		4480	33
	4		3453	7		1152	6		4605	7		3157	23
	5		10349	22		3499	19		13848	21		292	2
	6		2125	4		925	5		3050	5		1233	9
	7		5164	11		1889	10		7053	11		1767	13
Disability pension during follow-up			4371	9		961	5		5332	8		877	6
Follow-up time for disability pension		9.26 (2.80)			6.85 (1.31)			8.59 (2.70)			9.46 (1.56)		
No. of sickness absences during the previous year		0.20 (0.48)			0.19 (0.48)			0.20 (0.48)			0.12 (0.38)		
	0		39659	83		15349	84		55008	84		12067	89
	1		6477	14		2360	13		8837	13		1263	9
	2		1188	2		456	2		1644	2		174	1
	3		201	0		85	0		286	0		23	0
Self-rated health		1.93 (0.89)			1.83 (0.86)			1.90 (0.88)			1.76 (0.80)		
No. of chronic diseases		0.42 (0.66)			0.43 (0.66)			0.43 (0.66)			0.37 (0.61)		
	0		27783	58		10954	60		38737	59		9229	68
	1		11259	24		4734	26		15993	24		3415	25
	2		2594	5		988	5		3582	5		665	5
	3		449	1		188	1		637	1		85	1
BMI		25.02 (4.04)			25.11 (4.20)			25.04 (4.09)			24.83 (3.92)		
	< 18.5		571	1		251	1		822	1		206	2
	18.5–24.99		25989	55		9690	53		35679	54		7603	56
	25–29.99		14613	31		5628	31		20241	31		4351	32
	30 +		5249	11		2169	12		7418	11		1306	10
Smoking			8036	17		3485	19		11521	18		3343	25
Alcohol consumption		4.90 (5.72)			4.92 (5.77)			4.9 (5.74)			—		
Inactivity			9236	19		3454	19		12690	19	—		
Wake up several times per night		2.85 (1.61)			2.77 (1.59)			2.83 (1.61)			2.45 (1.20)		
GHQ		2.02 (0.45)			1.99 (0.44)			2.01 (0.45)			—		
Relational justice		3.63 (0.95)			3.72 (0.95)			3.65 (0.95)			—		
Procedural justice		3.02 (0.86)			3.06 (0.85)			3.03 (0.86)			—		
Participatory safety		3.59 (0.88)			3.59 (0.88)			3.59 (0.88)			—		
Support for innovation		3.14 (0.93)			3.13 (0.92)			3.14 (0.93)			—		
Vision		3.83 (0.66)			3.82 (0.66)			3.83 (0.66)			—		
Task orientation		3.33 (0.75)			3.34 (0.75)			3.33 (0.75)			—		
Social capital at work place		3.58 (0.76)			3.61 (0.76)			3.59 (0.76)			—		
Job strain			7623	16		2746	15		10369	16	—	2475	18
Effort-Reward imbalance			35132	74		15999	88		51131	78	—		
Shift work			15528	33		6393	35		21921	33	—		
Night shift			8393	18		3629	20		12022	18		1442	11

**Table 2:** Number of granted disability pensions per ICD-10 diagnosis group.

Diagnosis group	Development cohort	Validation cohort
Musculoskeletal (M)	2435	472
Mental health (F)	1270	293
Neoplasm (C)	362	96
Circulatory (I)	316	100
Nervous system (G)	307	96
Injuries (S)	129	43
Other	513	117

### Development of prediction score from all available data

We used parametric survival analysis to model the risk of work disability. Based on Akaike’s Information Criterion and graphical evaluation, lognormal distribution showed the best fit for modeling the baseline hazard function, and all models were therefore fitted with the lognormal distribution.

**Figure 2:**
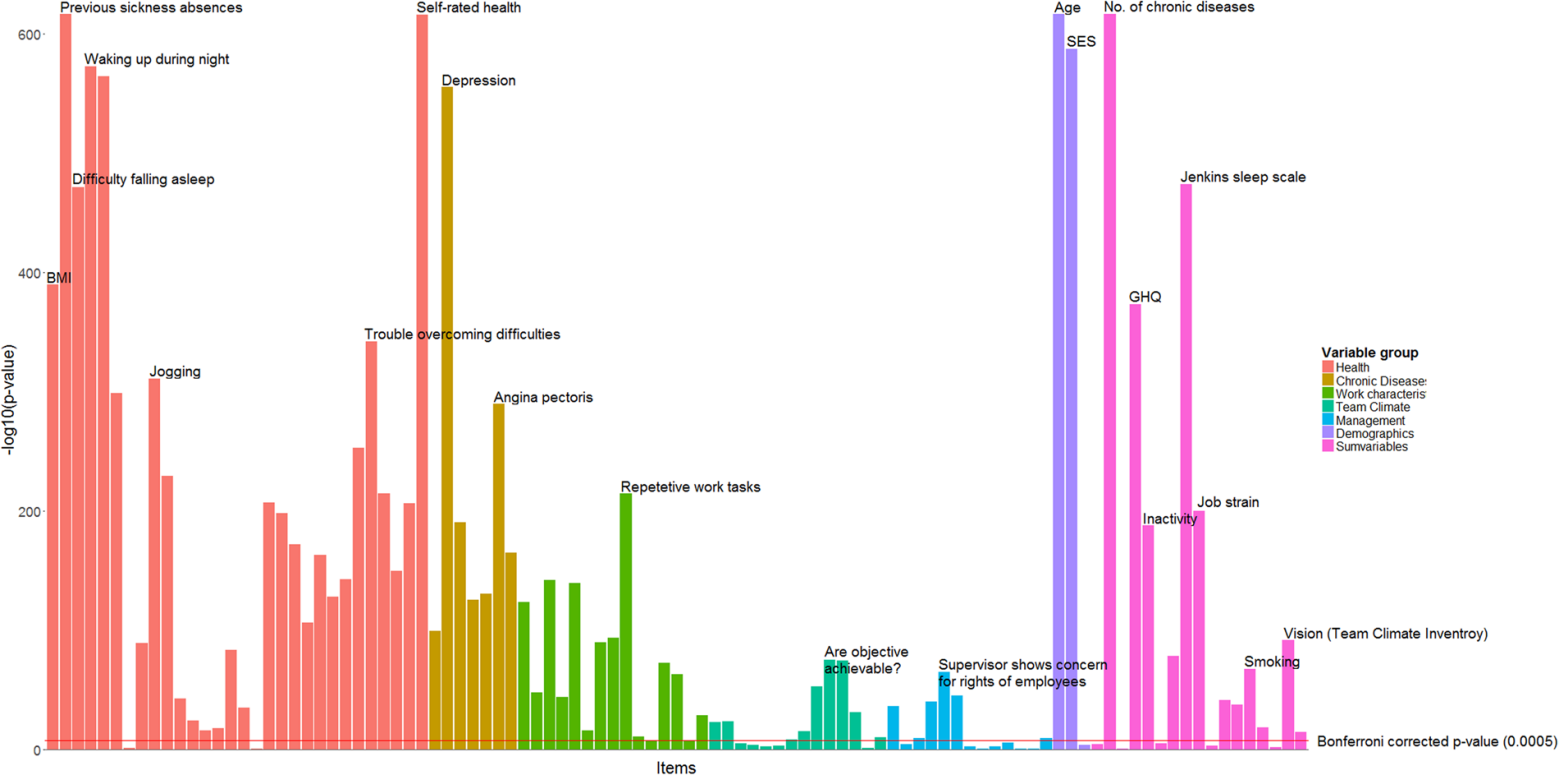
Bivariate association between predictor items and work disability. Items are grouped as described in the method section. All items included in the final model are labeled. Also labeled are most strongly associated items from each group, as well as other items that stand out.

The unadjusted bivariate associations between risk factors and work disability are illustrated in the Manhattan plot of Fig. 2. Nearly all variables were significantly associated with work disability. Age, socioeconomic position, health and disease-related items had the strongest associations. Many of the items related to work characteristics, team climate, and management were associated with work disability, but not as strongly as health-related items. In a redundancy analysis, social capital at work could be predicted with high accuracy with all other available variables and was therefore excluded from all further analysis. There were no other redundant variables.

The full model including all the non-redundant variables explained 21.7% of the variance in work disability. We then used backward stepwise regression analyses to obtain a more parsimonious prediction model, and found that almost all the variance (>99%) of the full model was captured by 8 variables: age, self-rated health, the number of long sickness absences in the previous year, socioeconomic position, chronic illnesses, sleep disturbance as indicated by the Jenkins scale, BMI and smoking. To determine which specific items of the Jenkins scale predicted work disability, we specified a new full model that included all the individual items instead of the summary variable. Only the item assessing difficulty falling asleep remained as a robust predictor and thus replaced the Jenkins scale in the parsimonious prediction model. Appendix 2 shows coefficients for the full model with all individual items from Jenkins scale, and the final model with 8 predictors. Adding specific chronic conditions, instead of the composite of having a history of any chronic disease, in the model as predictors did not improve the variance explained.

To refine the parsimonious prediction model to a multifactorial prediction score that allows linear and non-linear associations between the predictors and work disability, all the 8 continuous predictors were transformed into categorical variables. We categorized age into 5-year bands from 35 to 55 years plus a category of over 55 years. BMI was categorized according to the WHO classification^22^ into underweight (BMI < 18.5 kg/m^2^), normal weight (BMI = 18.5–24.9 kg/m^2^), overweight (BMI = 25–29.9 kg/m^2^) and obese (BMI ≥ 30 kg/m^2^). All the 6 other predictors were categorized according to their response options. We then fitted a model with the 8 categorical predictors (Table 3). While most of the associations between the predictors and work disability were linear or nearly linear, this was not the case for BMI, as both obesity and underweight were associated with an elevated risk of work disability. To assess internal validity, we fitted the 8-factor model separately for the two subsamples of FPS. The hazard ratios were similar in both subsamples in addition to the final combined sample, suggesting that the model was internally valid and reproducible (Table 3). As a sensitivity analysis, we also compared the hazard ratios of the final prediction score from the three best performing parametric models to those from Cox regression model. The log-normal model was again the closest match to the Cox model (Appendix 3).

**Table 3:** Hazard ratios and confidence intervals for predicting work disability in 10 years.

Predictor		FPS 2000			FPS 2004			FPS 2000+2004		
		n	HR	95% CI	n	HR	95% CI	n	HR	95% CI
Age										
	< 35	8024	1.00	—	7082	1.00	—	13141	1.00	—
	35–39	6161	1.23	(1.11, 1.34)	5544	1.42	(1.21, 1.63)	9017	1.26	(1.16, 1.36)
	40–44	7948	1.54	(1.44, 1.64)	7396	1.57	(1.37, 1.77)	10943	1.55	(1.46, 1.64)
	45–49	8517	2.45	(2.35, 2.55)	8637	2.21	(2.02, 2.40)	11275	2.42	(2.33, 2.50)
	50–54	9227	3.54	(3.44, 3.64)	8506	3.86	(3.67, 4.05)	11554	3.62	(3.54, 3.71)
	55+	7648	4.47	(4.37, 4.57)	9848	5.26	(5.07, 5.46)	9845	4.67	(4.58, 4.76)
Self-rated health										
	1 = Good	18029	1.00	—	17642	1.00	—	25909	1.00	—
	2	17105	1.26	(1.20, 1.31)	16819	1.24	(1.11, 1.37)	23424	1.26	(1.21, 1.32)
	3	10218	1.70	(1.64, 1.77)	10430	1.88	(1.75, 2.02)	13640	1.74	(1.68, 1.80)
	4	1995	3.02	(2.93, 3.11)	1974	3.47	(3.28, 3.67)	2591	3.11	(3.03, 3.19)
	5 = Poor	178	4.53	(4.32, 4.73)	152	5.50	(4.96, 6.04)	211	4.56	(4.37, 4.76)
No. of sickness absences during the previous year										
	0	39659	1.00	—	38851	1.00	—	55008	1.00	—
	1	6477	1.53	(1.48, 1.58)	6617	1.60	(1.49, 1.72)	8837	1.54	(1.50, 1.59)
	2	1188	2.10	(2.01, 2.19)	1257	2.23	(2.04, 2.41)	1644	2.10	(2.02, 2.18)
	3	201	3.26	(3.07, 3.45)	292	2.94	(2.57, 3.30)	286	3.11	(2.94, 3.27)
Socioeconomic position										
	1	1292	1.00	—	1310	1.00	—	1616	1.00	—
	2	12498	1.00	(0.85, 1.15)	12412	1.38	(0.94, 1.82)	17556	1.05	(0.90, 1.19)
	3	12462	1.31	(1.17, 1.46)	13235	1.78	(1.34, 2.21)	17861	1.37	(1.23, 1.51)
	4	3486	1.32	(1.16, 1.48)	3344	1.77	(1.31, 2.22)	4631	1.38	(1.23, 1.53)
	5	10427	1.66	(1.51, 1.80)	9652	2.23	(1.79, 2.66)	13936	1.73	(1.59, 1.87)
	6	2146	1.63	(1.47, 1.79)	2237	2.06	(1.60, 2.51)	3073	1.70	(1.54, 1.85)
	7	5214	1.84	(1.69, 1.99)	4827	2.39	(1.95, 2.83)	7012	1.93	(1.79, 2.08)
Chronic illness										
	0	14569	1.00	—	13668	1.00	—	20230	1.00	—
	1		1.25	(1.21, 1.31)		1.18	(1.08, 1.37)		1.25	(1.21, 1.32)
	2		1.55	(1.48, 1.77)		1.55	(1.40, 2.02)		1.56	(1.50, 1.80)
	3		1.52	(1.43, 3.11)		1.41	(1.22, 3.67)		1.71	(1.58, 3.19)
Trouble falling asleep										
	1 = Never	22031	1.00	—	21130	1.00	—	30353	1.00	—
	2	13202	1.01	(0.96, 1.07)	13125	1.03	(0.92, 1.15)	18367	1.03	(0.98, 1.08)
	3	6126	1.11	(1.05, 1.17)	6138	1.00	(0.86, 1.14)	8423	1.08	(1.02, 1.14)
	4	4241	1.17	(1.11, 1.24)	4580	1.07	(0.93, 1.22)	5977	1.14	(1.07, 1.20)
	5	673	1.20	(1.06, 1.34)	747	1.10	(0.81, 1.39)	961	1.22	(1.10, 1.35)
	6 = Almost every night	1252	1.25	(1.15, 1.35)	1297	1.27	(1.06, 1.48)	1694	1.25	(1.16, 1.34)
BMI										
	18.5–24.99	26575	1.00	—	24769	1.00	—	36512	1.00	—
	25–29.99	15002	1.07	(1.02, 1.11)	15662	1.05	(0.95, 1.15)	20792	1.07	(1.03, 1.11)
	< 18.5	577	1.19	(1.00, 1.39)	500	1.38	(0.98, 1.78)	837	1.19	(1.01, 1.37)
	30+	5371	1.20	(1.14, 1.26)	6086	1.15	(1.02, 1.27)	7634	1.19	(1.14, 1.25)
Smoking										
	No	39099	1.00	—	38963	1.00	—	53692	1.00	—
	Yes	8426	1.16	(1.11, 1.21)	8054	1.23	(1.13, 1.34)	12083	1.18	(1.13, 1.22)

All coefficients are derived using imputed samples.

Abbreviations: FPS 2000, Finnish Public Sector Study, 2000 survey and linkage to electronic health records; FPS 2004, Finnish Public Sector Study, 2004 survey and linkage to electronic health records; HR, Hazard ratio; 95% CI, 95% confidence intervals.

**Figure 3:**
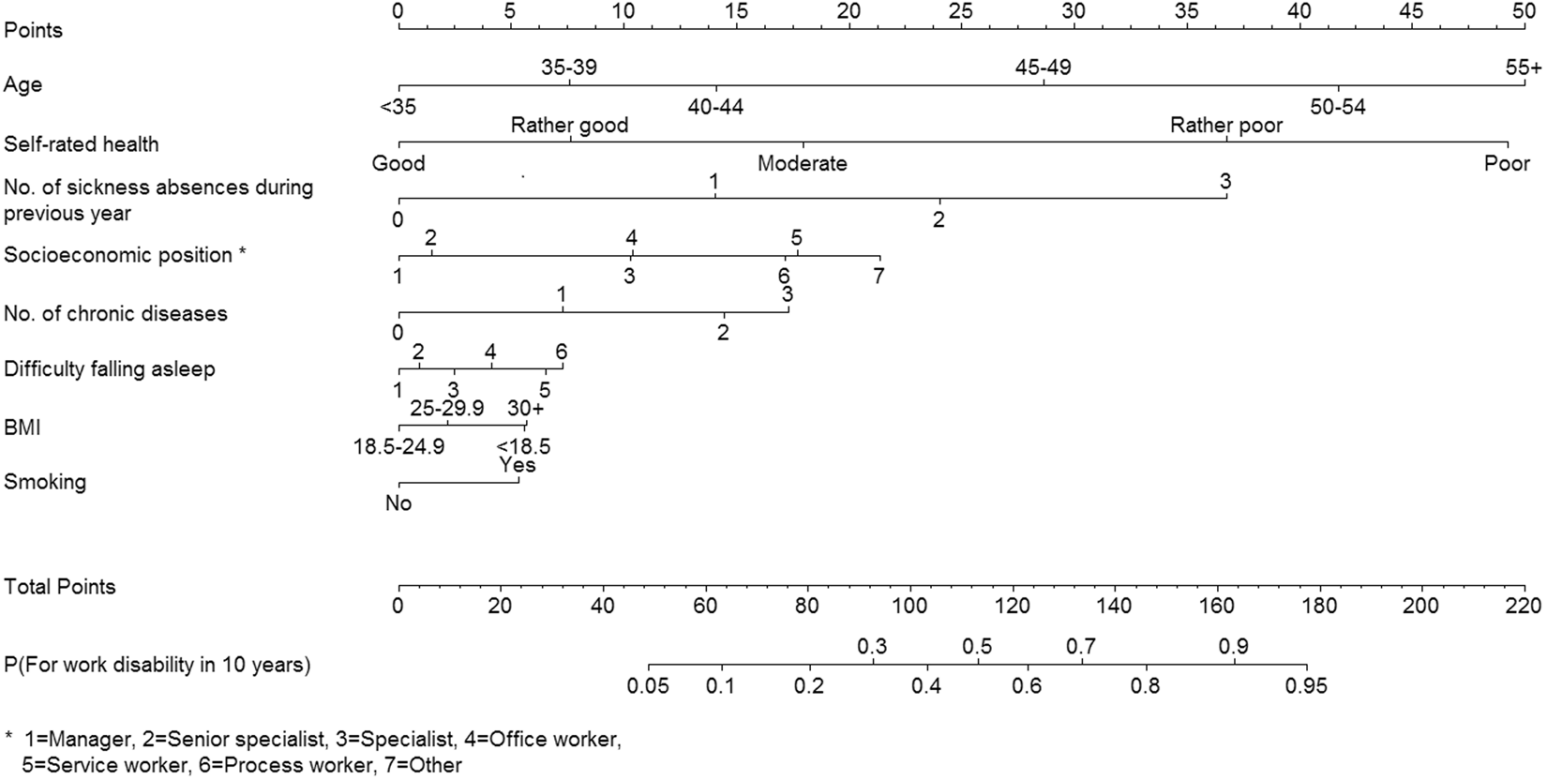
Nomogram for the final risk prediction model.

**Figure 4:**
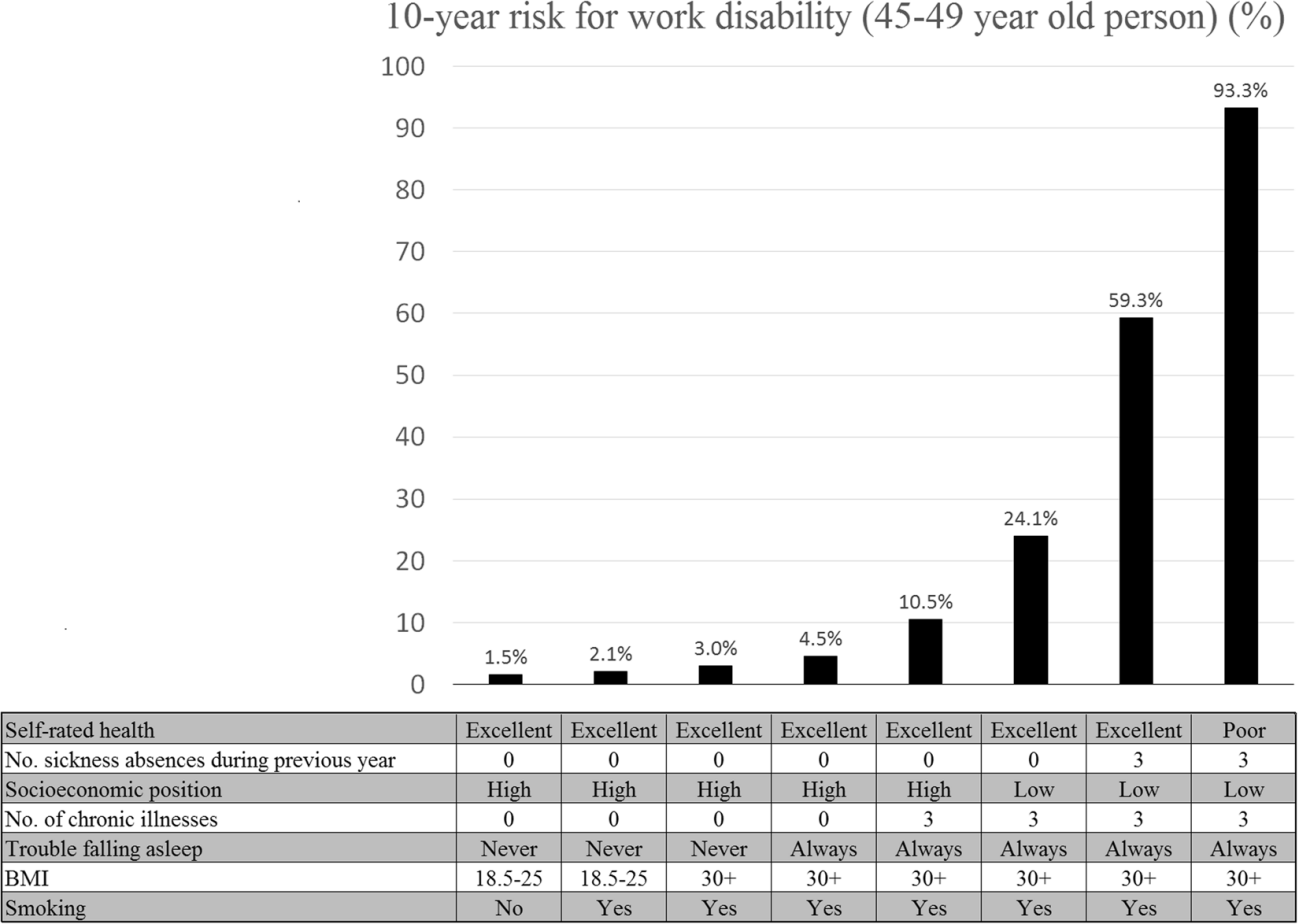
Risk for disability pension in 10 years for a person aged 45–49 with increasing number of risk factors.

Figure 3 shows a nomogram of the weights and points of the 8-factor prediction score allowing estimation of an individual’s risk manually without having to resort to the actual formula (for details of the nomogram, see Appendix 4). To illustrate risk accumulation, Fig. 4 shows how each additional risk factor increases the absolute risk of disability starting from a risk profile with minimum risk factors at age 45–49 years. In that group, the risk of disability was 1.5%. The risk increased after each additional risk factor, being 2.1% if the person smoked, 3.0% if also obese, 4.5% if having trouble falling asleep, 10.5% with prevalent chronic disease, 24.1% if socioeconomic position was additionally low, and 59.3% with a history of 3 or more sickness absence during the preceding 12 months. For a person with all these risk factors plus poor self-rated health, the risk of work disability during the next 10 years was as high as 93.3%.

### Validation of the 8-factor prediction score

Further internal validation in the combined FPS sample supported the high predictive performance of the score. The C-index was 0.840 (95% confidence interval: 0.834 to 0.845) and internal validation with bootstrapping indicated minimal over-fitting with the optimism-corrected C-index being 0.838.

External validation was done using data from the HeSSup survey which did not include exactly the same questions as those used in the survey of the development sample (for the wordings of the questionnaire items, see Appendix 1). The main differences were in the questions on socioeconomic position and sleep problems. In HeSSup, socioeconomic position was assessed by educational level (1 = university degree, 7 = no vocational degree), and sleep by the question “How well do you usually sleep?” (1 = well, 4 = poorly). Responses for educational level in the validation cohort were treated as equal to those of socioeconomic position in the development cohort. As the response format for the sleep question was a 4-rather than 6-point scale, the responses were rescaled to a 6-point scale using rounding to the closest integer. Despite these minor differences, the 8-item score yielded a high C-index of 0.828 (95% confidence interval: 0.815 to 0.841) in the independent validation sample.

**Figure 5:**
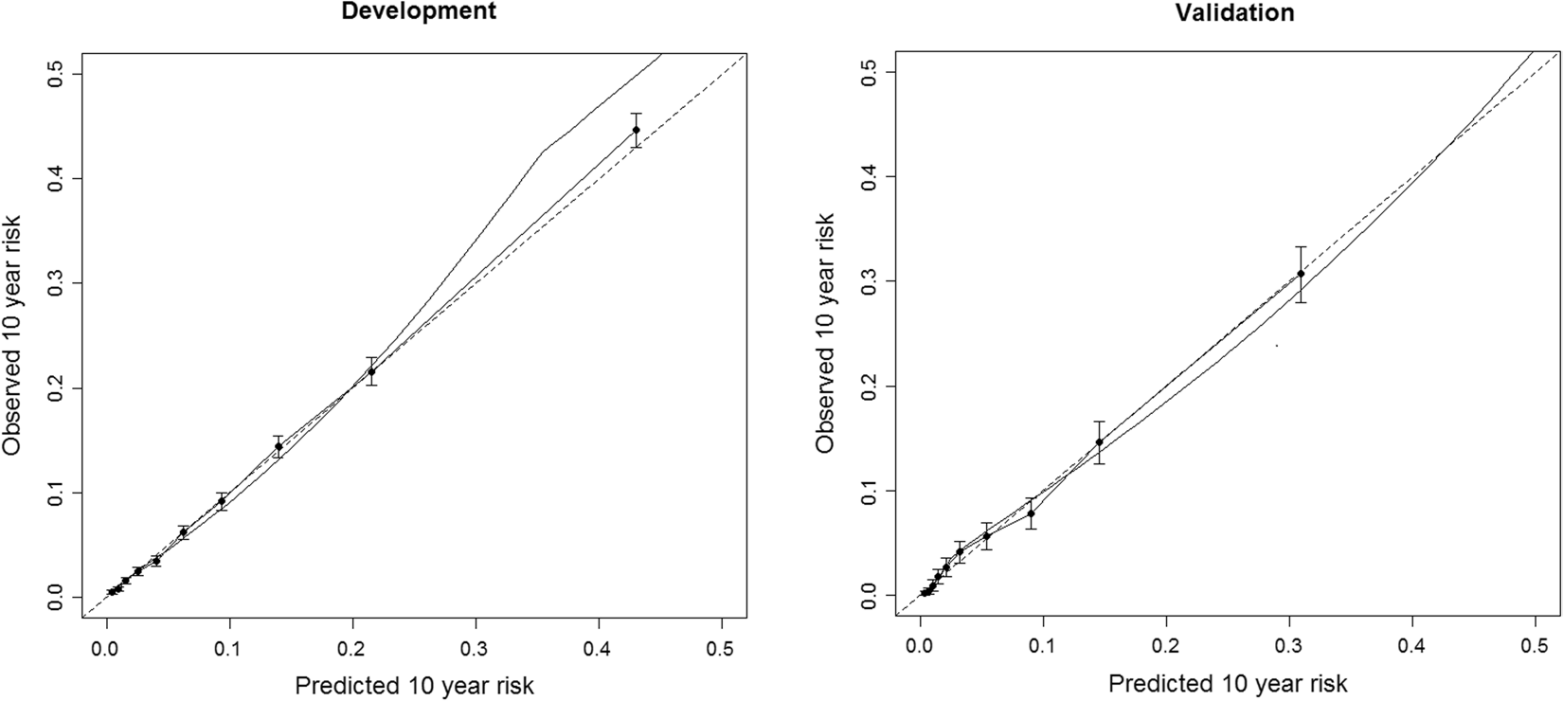
Calibration plots for FPS (development sample) and HeSSup (validation sample). Dotted line is the ideal calibration, solid black line is the fitted polynomial spline, and the dots are decile risk groups and their 95% confidence intervals.

The absolute accuracy of the prediction score was then evaluated using calibration plots. As shown in Fig. 5, these plots suggested a high correspondence between the predicted and the observed risk both in the development and validation cohorts. For example, in the bottom two deciles of the score the predicted risks were 0.4% and 0.9% and the observed risks 0.5% and 0.8% in the development sample. In the validation sample, the corresponding predicted risks were 0.4% and 0.6% and the observed risks 0.2% and 0.4%, respectively. The predicted risk in the top decile was slightly overestimated: in development sample the predicted risk for that decile was 43.0%, whereas the observed risk was 44.6%. In the validation sample the corresponding risks for the same decile were 30.9% and 30.7%, respectively.

### Development of an alternative score using work-related variables

**Figure 6:**
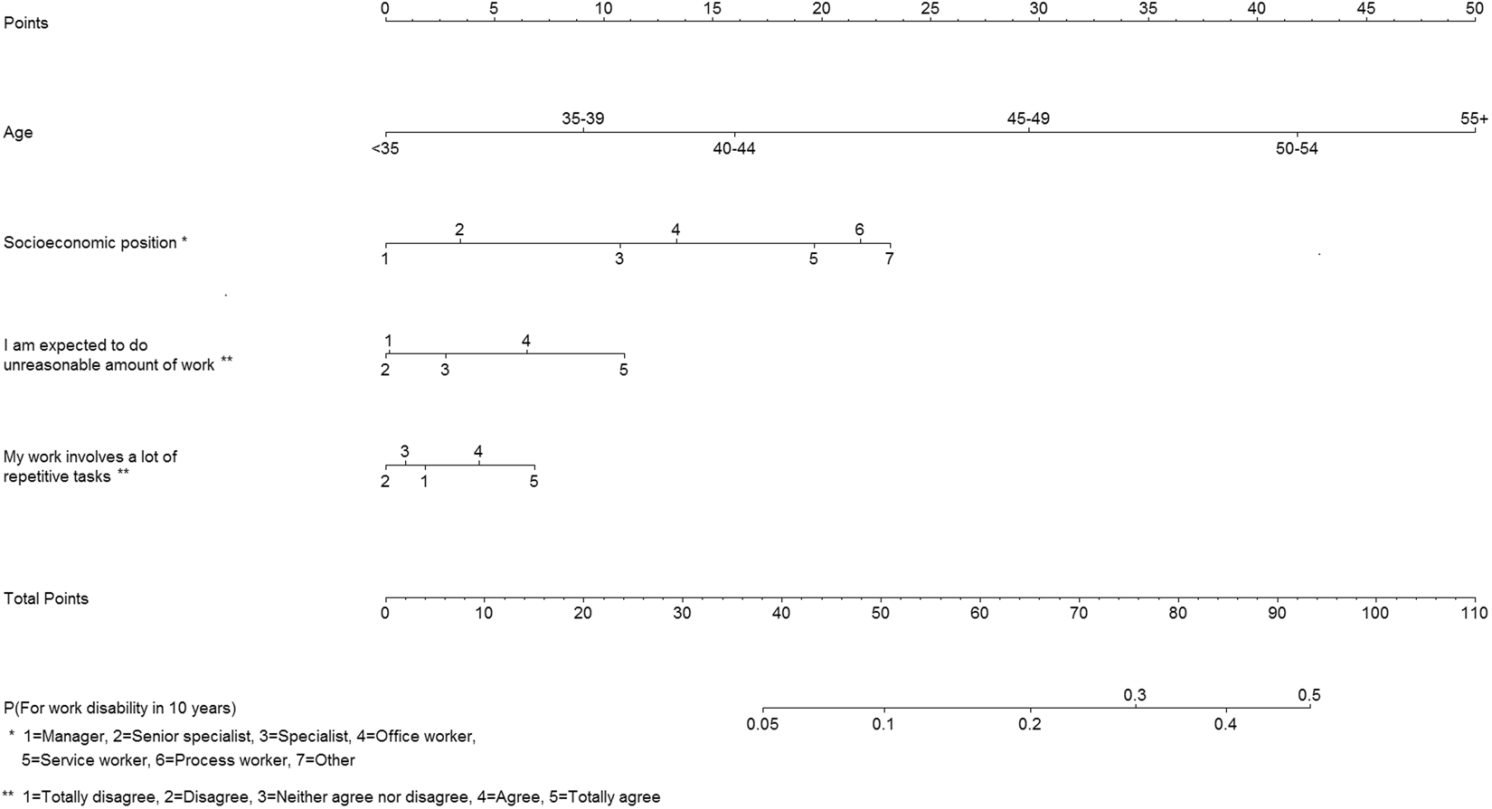
Nomogram for the alternative model with two work-related items.

As workplace questionnaire surveys often include only questions on basic demographic characteristics and work-related issues, we created another prediction model using only age, sex, socioeconomic status, and all work-related factors as candidate variables. Model development was based on following the same procedure as with our main prediction model. Factors explaining 99% of the variance of full model were age, socioeconomic position and job strain scale. C-index in internal validation was 0.784 (95% confidence interval: 0.778 to 0.789). External validation with the data from the HeSSup survey yielded a C-index of 0.774 (95% confidence interval: 0.760 to 0.789) (for model coefficients, see Appendix 5). Other work-related factors, such as shift work and scales of effort-reward imbalance at work, procedural and relational justice, team climate inventory, including participatory safety, support for innovation, vision, and task orientation, did not improve prediction. We then examined whether the predictive ability of work-related score can be improved by disaggregating the job strain subscales and developing a prediction algorithm using items that are most strongly related to work disability. Using this procedure, one item from both the job demand (“*I am expected to do unreasonable amount of work*”) and job control (“*My work involves a lot of repetitive tasks*”) scales were chosen. The C-index for a model including these 2 items in addition to age and socioeconomic position was 0.788 (95% confidence interval: 0.782 to 0.793) in internal validation and 0.780 (95% confidence interval: 0.765 to 0.794) in external validation. Figure 6 shows the related nomogram (for coefficients and calibration plots, see Appendix 6).

Finally, we developed a prediction model from a pool of demographic variables and all work-related items (rather than scales). In this analysis, a parsimonious model included the following 4 statements or questions in addition to age and socioeconomic position: “*I am expected to do unreasonable amount of work*”, “*My work involves a lot of repetitive tasks*”, “*I have a say in the tasks included in my work*”, *and* “*Your supervisor shows concern for your rights as an employee*”. The C-index did not improve (0.789; 95% confidence interval: 0.783 to 0.795). This model could not be externally validated as data on one of the items were not available in the validation cohort.

### Internet-based tool to estimate the risk of work disability

As way to disseminate these findings, we developed a web-based questionnaire for the calculation of a personalized 10-year work disability risk based on the validated 8-factor score and the validated score using work-related variables (available as an online appendix in the journal’s web-page: https://doi.org/10.1038/s41598-017-13892-1).

## Discussion

We developed and validated, internally and externally, a new risk prediction score for work disability. The score is based on eight variables: age, self-rated health, number of sickness absences in the previous year, socioeconomic position, presence of chronic illnesses, having difficulty falling asleep, BMI, and smoking. The score performed well in the development cohort and in the independent validation cohort, with discriminative ability of 0.84 and 0.83, respectively. In addition, the predicted risks were in accordance with the observed risks through all risk deciles in both cohorts and the score captured a wide range of absolute risks. In the age group of 45 to 49-years, for example, absolute risk of work disability was 1.5% for a person with optimal risk profile and 93.3% for one with all measured risk markers. Alternative risk prediction scores, based on demographic characteristics and work-related factors, had more modest predictive abilities, although the observed c-statistics in the range of 0.70 to 0.80 are still considered to indicate acceptable discrimination.

All variables included in the prediction scores have previously been associated with disability pension. Poor self-rated health, for example, predicted disability pension from any cause even after controlling for various health and working conditions related factors^23^. Earlier findings have also identified age and a history of sickness absences as predictors of disability pension^24,25^. Similarly smoking and obesity have been associated with an increased risk of early exit from workforce^9,26^. The predictive performance of our 8-factor score, which is applicable across occupations or branches of occupation, is comparable to that for the 15-factor soldiers’ disability risk, the C-index being 0.828 in the present study and 0.860 for soldiers’ score in a US army population. The predictive performance of our score was at the same level as those for standard prediction scores for chronic conditions that are currently recommended internationally for disease prevention in clinical practice^27,28^. The C-statistics for the widely used U.S. Framingham Cardiovascular Risk Scores^29,30^ the European equivalent, SCORE predictor^14^, and the UK QD-Score for prediction of diabetes^15^, for example, have been slightly above and below 0.8. This supports the feasibility of our score in real-life settings.

There is a large body of research on work characteristics and work disability. In the present study, a wide range of validated questionnaire scales on work-related stress, team working, leadership and organizational justice were included in the baseline assessment^31–34^. Interestingly, these work characteristics were not retained in our final 8-factor prediction algorithm, suggesting that work characteristics may be more distal in the causal pathway and not as strongly predictive of an individual’s risk of work disability compared to more proximal risk factors such as health status, lifestyle habits and socioeconomic position. Of the multiple work-related concepts, job strain, the most widely used model of psychosocial work stress^32^ had better predictive capacity than more recent derived characteristics, such as effort-reward imbalance^34^, procedural and relational justice^35^, and team climate^36^.

It is important to make a distinction between risk prediction and targets of intervention to prevent work disability. The purpose of risk prediction in this context is to identify a group of people at increased risk of work disability. The items in the multifactorial score are not necessarily the best targets for prevention; indeed, they can even be risk markers (e.g., HDL-cholesterol is a component of the Framingham risk score for prediction of cardiovascular disease, but reduction of LDL-cholesterol is the main target of lipid-lowering treatments). Further research is needed to develop effective, cost-effective and safe measures to reduce the occurrence of work disability in groups at risk.

### Strengths and limitations

To the best of our knowledge, our 8-factor model is the first internally and externally validated multifactorial prediction score for work disability. That the variables included in the model can be easily assessed by health care professionals or by the individuals themselves facilitates the use of the score in multiple settings ranging from occupational and primary health care to web-based applications. Early detection of individuals at high risk is a precondition to effective targeted interventions to prevent work disability and a basis for developing cost-effective strategies to lengthening work career. Our validated score for 10-year prediction of work disability could serve as a screening tool for clinicians and occupational health professionals in identifying those at the greatest risk for work disability. Such instruments are needed in the process of work disability evaluation, which has been demonstrated to show high variability and low reliability^37^, especially in cases were no such instruments are used. Further research is needed to evaluate potential benefits and harms of the usage of the screening tool in diverse working populations and the feasibility of our score as a web-based tool to enable employees’ self-evaluation of their risk for work disability.

The present study has some limitations. Both the development and validation cohorts were based in Finland where ascertainment of work disability was possible with linkage to comprehensive records from the national pension register with virtually full coverage on all gainful employment^38^. However, the generalizability of our score should be tested in other settings. Even though the calibration of the prediction score proved to be high across the observed risk range, most of those risks were small −70% of the risks in the development cohort were smaller or equal to 10% risk of work disability in 10 years. A lifetime prediction model would identify are large proportion of people at high risk, although the predictive validity of short scores for longer prediction windows is likely to be lower.

Also, the validation cohort differed from the development cohort on the assessment of socioeconomic position and sleep disturbances. In the development cohort socioeconomic position was measured using current occupational status, whereas in the validation cohort the measure was occupational education. The measures might capture different parts of the underlying concept. However, the validation study showed that both measures of socioeconomic position performed equally well. Similarly, the questions on sleep relate to slightly different features of sleep in the two cohorts, although the weight given to sleep problems in the model was modest and therefore such differences are unlikely to affect the results in a significant way.

Finally, the process of evaluating work disability by local authorities may affect the generalizability of our findings. Although work disability is defined by impairment, unlike commonly used clinical outcomes, such as adverse cardiac/cerebrovascular events and mortality, receipt of a disability pension is additionally dependent on non-medical factors, such as disability pension regulations, the work environment, the nature of the job, and the extent to which the workplace is prepared to accommodate the disability. There is a need for further research to examine the predictive validity of our score across populations and countries with different labor market regulations.

### Conclusions

We developed and validated internally and externally a new 10-year prediction score for work disability which included information from sociodemographic and lifestyle factors. The 8-item score showed high predictive performance and discriminative ability in two large independent samples from Finland. An alternative score including only demographic and work-related items had slightly lower discriminative ability. As easy-to use scores, these measures could be valuable screening tools for identifying those at most risk of health-related early exit from the workforce, but further research is needed on benefits and harms and generalizability across different labor market settings.

## Data and Methods

### Study design and participants

We used individual-level data from the Finnish Public Sector study (FPS) for the development of the prediction score (‘development’ cohort). We then validated this score in an independent, population-based cohort study, the Health and Social Support study (HeSSup) (‘validation’ cohort). Both studies have been described in detail elsewhere^19–21^.

Briefly, the participants of FPS were employees in the municipal service of 10 Finnish towns and 21 public hospitals, including a range of professions (city mayors, teachers, cleaners, construction workers, and so on). The study was approved by the Ethic committee of the Hospital District of Helsinki and Uusimaa. A wide set of potential predictors of work disability was assessed using a standardized survey in 2000–2002 when 48,598 participants responded (response 68%) and in 2004 when 48,076 responded (response 66%). We excluded participants who were on a long-term sickness absence (≥90 days), disability pension, or retired at the time of responding (N = 478 in 2000–2002 and N = 578 in 2004) as well as those missing the personal identification number used for data linkage (N = 595 in 2000–2002 and N = 481 in 2004). We also excluded those participants from the second survey who had taken part in the first survey (N = 28,767). Thus, the sample of the 2000–2002 survey included 47,525 employees (subsample 1), and the 2004 survey included 18,250 employees (subsample 2). We combined the subsamples to form the final development sample. The combined sample included 65,775 employees. We linked these participants to the electronic records of work disability, sickness absence, statutory retirement and mortality registers until the end of 2011 using personal identification numbers assigned to all Finnish citizens, an exercise that was successful for all participants. A flow chart of the sample selection is shown in Fig. 1.

HeSSup is a prospective cohort study that began in 1998^20^ and targeted a population sample representative of the Finnish population in four age groups: 20–24, 30–34, 40–44, 50–54 years at baseline. The study was approved by the Turku University Central Hospital’s Ethics Committee. A total of 25,898 people participated in the baseline survey, of whom 14,683 were employed. Of them, we excluded those who did not give consent to link their responses to electronic records of work disability (N = 1055), or were on a long-term sickness absence ( ≥ 90 days), disability pension or retired at the time of responding or died before the start of the follow-up (N = 101). Thus, the final validation data included 13,527 participants who were linked to records of disability pension, sickness absence, retirement and death until 31 December 2008.

All participants provided written informed consent. Further, this study and its methods were conducted according to the guidelines of the Helsinki declaration.

### Measurement of predictors of work disability

The FPS survey included 82 questions assessing participants’ sociodemographic characteristics, health status, lifestyle, and working conditions. For a full list of the items see Appendix 1. These questions relate to the following 23 single- or multi-item candidate predictors.

*Sociodemographic factors* were derived from employers’ registers and included sex, age, and socioeconomic position. Socioeconomic position was derived from the participants’ job titles using the International Standard Classification of Occupations (ISCO). The ISCO has ten categories ranging from 1 (managers) to 9 (elementary occupations) with a separate category for armed force occupations. With categories 6–8 (skilled agricultural workers, craft and trade workers, plant and machine operations) referring to similar skill level occupations, and relatively few participants falling into those categories, they were combined to form a single “process worker” category. Further, none of the participants were employed in armed forces, so the final measure for socioeconomic position was (1–7): 1 = manager/higher official, 2 = senior specialist, 3 = specialist, 4 = office worker, 5 = service worker, 6 = process worker, and 7 = other/elementary occupations.

#### Chronic diseases

Participants were asked to report physician-diagnosed diseases from a list of common ailments. We matched the diseases with the 30 leading contributors of global disability-adjusted life years^3^. The list of diseases included bronchial asthma, myocardial infarction, angina pectoris, cerebrovascular diseases, migraine, depression, and diabetes. Diseases that were among the leading causes, but not asked in the survey, included diseases such as sense organ diseases, lung cancer, and a range of severe communicable diseases. We formed a new variable for chronic diseases by summing the number of reported chronic diseases. The number ranged from 0 to 7 with 180 individuals reporting 4 or more (who were collapsed to the category of “3 or more”).

#### Health status and health behaviors

Self-rated health was assessed using a 5-point scale (1 = good, 2 = rather good, 3 = moderate, 4 = rather poor, 5 = poor). The 12-item general health questionnaire (GHQ) was used to assess psychological distress^39^. Responses for GHQ were given on a 4-points Likert scale (i.e., 1 = better than usual, 4 = much worse than usual) and a mean response was used in analyses. Sleep problems were assessed using the four item Jenkins sleep problem scale^40^ and responses were given on a 6-point Likert scale (i.e., 1 = never, 6 = almost every night). Alcohol consumption was assessed with question on how much beer, wine, and spirits the participants consumed in a week. The average for each beverage was transformed into units of alcohol per week (range from 0 to 226 units a week and a median of 3) and the variable was truncated from the top to interquartile range (IQR) + IQR*3, so that the range was from 0 to 21. Other measures included smoking (0 = non-smoker/former smoker, 1 = current smoker) and leisure-time physical inactivity (0 = active, 1 = sedentary). Self-reported height and weight were used to calculate body mass index (BMI; weight in kilograms divided by height in meters squared). All spells of sickness absences longer than 9 days during the previous year were obtained from electronic health records curated by the Social Insurance Institution of Finland. The number of sickness absences had a range from 0 to 5 with 68 individuals reporting 4 or 5 spells of absence. Observations measuring 4 or over were recoded as 3, resulting in a range from 0 to 3.

#### Work-related factors

The FPS questionnaire included questions about shift work (0 = no, 1 = yes), night shift (0 = no, 1 = yes), and multi-item scales of job demand (a 3-item scale), job control (a 6-item scale), job effort (1 items) and job reward (a 3-item scale) with a 5-point Likert scale as the response format. The partial scales used for job demand and job control have been shown to be in high agreement with the full scales^41^. The mean score of job demand and job control scales were used to construct a measure of job strain^42^. Job strain was defined as 1, if job demand was above the median and job control below the median, and 0 otherwise. For job rewards we averaged the responses and the ratio of effort to rewards was used to form a dichotomized measure of effort-reward imbalance at work, with value 1 if the ratio was over 1, and 0 otherwise^43^.

#### Team work and management

These measures included procedural (7 items) and relational justice (6 items)^35^, and a short version of team climate inventory (TCI)^44^ which includes four subscales labelled as *participatory safety* (4 items), *support for innovation* (3 items), *vision* (4 items), and *task orientation* (3 items). Also included was a scale for social capital at work (8 items), which consisted of a combination of items from the four TCI scales. Responses to the items of procedural and relational justice, and TCI scales were given using a 5-point Likert scale. The mean of the responses on each scale were used in analysis.

### Ascertainment of work disability

In Finland, earnings-related pension security covers almost all gainful employment. We obtained records of the starting date and type of all pensions from the national register of the Finnish Centre of Pensions^38^. The Finnish Centre of Pensions has a statutory obligation to keep records of pensions and produce statistics from all registries and pension providers in Finland. A full disability pension can be granted for a person whose capacity for work is severely impaired by at least 60% due to a disease, injury or handicap. These records have been widely used in a research context^11,45,46^. All the employees in both studies were insured in some pension scheme and therefore disability pension records were available for all study participants. The examined outcome was full time disability pensions that could be temporary or permanent.

### Statistical Analysis

We combined the two FPS subsamples to form the development cohort of 65,775 employees. We imputed missing data (2.8% of all observations) on predictors using single imputation with predictive mean matching^47^.

We used Akaike’s Information Criterion and graphical evaluation to compare how well different parametric distribution fit the baseline hazard function. Then we examined bivariate associations between all the predictor items and summary variables using parametric survival models. Reducing the number of predictors based on unadjusted association with the outcome is not recommended by the TRIPOD statement, as it may lead to rejection of important predictors based on nuances in the data^48^. Therefore we included all the variables in the following analyses, even if there was no significant association in the bivariate analysis. Before further model specification, we ran a redundancy analysis to exclude variables that could be readily predicted using all other variables. Following the procedure of Hijazi and colleagues^49^, we specified a parametric survival model that included all 23 candidate variables as predictors (‘full model’). To obtain a more parsimonious model, we derived the model-predicted work disability risks for each individual based on the full model. We then used backward stepwise ordinary least squares regression in which we predicted risks derived from the full model. In the first step all original variables used in the full model were retained in the model, and R^2^ equals unity by design. We then removed as many variables as possible, while retaining a R^2^ value close to 100%. The satisfactory level of R^2^ obtained this way is a balance between a model that explains most of the variance of the full model (more variables leads to higher R^2^) and the number of variables retained in the final model (fewer variables leads to a more parsimonious model). If any summary variables were left in the model after the backward stepwise regression analysis, we defined the full model again with the summary variable(s) broken down to individual items. We then repeated the analysis as described above. This allowed us to identify the individual items that explained most of the variance of the full model and enabled us to form a parsimonious model. The predictors that were left in the model after these steps formed the final multifactorial prediction score – again, a parametric survival model.

The performance of the final prediction score was evaluated using Harrell’s C-index, which is the concordance between predicted and observed survival^47^. To estimate the degree of overfitting, the model was first internally validated using the bootstrapping method^48^, and then externally validated by assessing the performance of the score in the HeSSup cohort study. Calibration of the model - how accurately the predicted absolute risks correspond to observed absolute risks - was assessed using calibration plots, separately for the development data and validation data. All analyses were performed using R 3.2.3 (packages: mice, rms and Hmisc).

### Data sharing

Statistical syntax and anonymized data on predictors are available for bona fide researchers. Individual-level data on disability pensions are owned by the Finnish Institute of Occupational Health and University of Helsinki; no sharing of those data are permitted.

## Electronic supplementary material

Supplementary information

Risk calculators
