# Author Correction: Development and validation of a risk prediction model for work disability: multicohort study

**DOI:** 10.1038/s41598-018-35363-x

**Published:** 2018-11-16

**Authors:** Jaakko Airaksinen, Markus Jokela, Marianna Virtanen, Tuula Oksanen, Jaana Pentti, Jussi Vahtera, Markku Koskenvuo, Ichiro Kawachi, G. David Batty, Mika Kivimäki

**Affiliations:** 1 Finnish Institute of Occupational Health, Helsinki, Finland; 2 Department of Psychology and Logopedics, Faculty of Medicine, University of Helsinki, Helsinki, Finland; 3 Department of Public Health, University of Turku, Turku, Finland; 4 Clinicum, Faculty of Medicine, University of Helsinki, Helsinki, Finland; 5 Harvard T H Chan School of Public Health, Boston, MA USA; 6 Department of Epidemiology and Public Health, University College London, London, UK

Correction to: *Scientific Reports* 10.1038/s41598-017-13892-1, published online 19 October 2017

The Supplementary material ‘Risk calculators’ that accompanies this Article contains an error in the ‘8-factor model’ tab, where “Wake up several times per night” should read “Difficulty falling asleep”.

