# Supplementary material for: Development and validation of a risk prediction model for work disability: multicohort study
Source: Sci Rep. 2017 Oct 19;7:13578. doi: 10.1038/s41598-017-13892-1 (PMC5648892; doi:10.1038/s41598-017-13892-1)
Supplement: Supplementary file 1 — Supplementary information [file 41598_2017_13892_MOESM1_ESM.pdf]

Development and validation of a risk prediction model for work disability: multicohort study

Jaakko Airaksinen[1*], Markus Jokela[2], Marianna Virtanen[1], Tuula Oksanen[1], Jaana Pentti[3,4], Jussi Vahtera[3], Markku Koskenvuo[4], Ichiro Kawachi[5], G. David Batty[6], Mika Kivimäki[1,4,6]

1 Finnish Institute of Occupational Health, Helsinki, Finland
2 Department of Psychology and Logopedics, Faculty of Medicine, University of Helsinki, Finland
3 Department of Public Health, University of Turku, Finland
4 Clinicum, Faculty of Medicine, University of Helsinki, Helsinki, Finland
5 Harvard T H Chan School of Public Health, Boston MA, USA
6 Department of Epidemiology and Public Health, University College London, London, UK

*Corresponding author: Dr Jaakko Airaksinen, Finnish Institute of Occupational Health, P.O. Box 40, FIN-00251 Helsinki, Finland. E-mail: jaakko.airaksinen@ttl.fi, Tel.: +358 46 922 0548

Appendix 1.

Table A. Variables, and items that those variables consist of, that were included in the full prediction model (Development cohort)

**Variables**	**Items**
Sex	
Age	
BMI	
	Height in cm
	Weight in kg
Socioeconomic position	Status in employment
Smoking	
	Do you smoke or have you smoked regularly (every day or almost every day)?
	Do you still smoke regularly?
Alcohol consumption	
	Have you ever had at least a glass of an alcoholic beverage?
	How many times a week you consume beer?
	…wine?
	…spirits?
	How many times have you passed out from drinking during the past year?
Inactivity	
	During the past year, how many hours in a week have you walked?
	…walked briskly?
	…jogged?
	…ran?
GHQ	
	In past weeks have you been able to concentrate?

	...loss of sleep over worry
	...playing a useful part
	...capable of making decisions
	...felt constantly under strain
	...couldn’t overcome difficulties
	…able to enjoy day-to-day activities
	...able to face problems
	...feeling unhappy and depressed
	...losing confidence
	...thinking of self as worthless
	…feeling reasonably happy
Chronic illness	
	Bronchial asthma
	Myocardial infarction
	Angina pectoris
	Cerebrovascular diseases
	Migraine
	Depression
	Diabetes
Self-rated health	
Jenkins sleep scale	
	How many times in the past 4 weeks have you had ...trouble falling a sleep
	…frequent awakenings during the night
	...trouble remaining asleep
	...feelings of fatigue and sleepiness despite receiving a typical night’s rest
No. of sickness absences in previous year	
Job strain	Job control

My work requires creativity
My work requires me to learn new things
My work involves a lot of repetitive tasks
I have a say in the tasks included in my work
My work requires highly developed skills
I have very little freedom to decide how I do my work

Job demand

My work requires a lot of effort
I am expected to do unreasonable amount of work
I have sufficient time to get my work done

Relational justice

Your supervisor considers your viewpoint
Your supervisor is able to suppress personal biases
Your supervisor provides you with timely feedback about the decision and its implications
Your supervisor treats you with kindness and consideration
Your supervisor shows concern for your rights as an employee
Your supervisor takes steps to deal with you in a truthful manner

Procedural justice

Procedures designed to… collect accurate information necessary for making decisions.
…provide opportunities to appeal or challenge the decision
…have all sides affected by the decision represented.
…generate standards so that decision could be made with consistency.
…hear the concerns of all those affected by the decision.
…provide useful feedback regarding the decision and its implementation
…allow for requests for clarification or additional information about the decision.

Participatory safety

People keep each other informed about work-related issues in the team
There are real attempts to share information throughout the team

	We have a "we are in it together" attitude
	People feel understood and accepted by each other
Support for innovation	
	People in this team are always searching for fresh, new ways of looking at problems
	In this team we take the time needed to develop new ideas
	People in the team co-operate in order to help develop and apply new ideas
Vision	
	To what extent do you think your team's objectives are clearly understood by other members of the team?
	How far are you in agreement with these objectives?
	To what extent do you think your team's objectives can actually be achieved?
	How worthwhile do you think these objectives are?
Task orientation	
	Are team members prepared to question the basis of what the team is doing?
	Does the team critically appraise potential weaknesses in what it is doing in order to achieve the best possible outcome?
	Do members of the team build on each other's ideas in order to achieve the best possible outcome?
Social capital at work place	Do members of the team build on each other's ideas in order to achieve the best possible outcome?
	People keep each other informed about work-related issues in the team
	We have a "we are in it together" attitude
	People feel understood and accepted by each other
	People in the team co-operate in order to help develop and apply new ideas
	Do members of the team build on each other's ideas in order to achieve the best possible outcome?
	Your supervisor treats you with kindness and consideration
	Your supervisor shows concern for your rights as an employee
	Your supervisor takes steps to deal with you in a truthful manner
Effort-Reward imbalance	

	Effort	
		How much of your skills and resources you invest in your work?
	Reward	
		Do you feel that you get value for money for your work?
		Do you feel that you get recognition and respect for your work?
		Do you feel that you get personal satisfaction of your work?
Shift work		
Night shift		

Table B. Items in the validation cohort

Sex		
Age		
Socioeconomic position	Highest achieved degree	
No. of sickness absences in during year		
Self-rated health		
No. of chronic diseases		
	Bronchial asthma	
	Myocardial infarction	
	Angina pectoris	
	Cerebrovascular diseases	
	Migraine	
	Depression	
	Diabetes	
BMI		
Smoking	Do you smoke?	
Sleep	How well you usually sleep?	
Night shift		
Job strain	Job control	
		My work requires creativity
		My work requires me to learn new things
		My work involves a lot of repetitive tasks
		I have a say in the tasks included in my work
		My work requires highly developed skills
		I have very little freedom to decide how I do my work
	Job demand	
		My work requires a lot of effort
		I am expected to do unreasonable amount of work
		I have sufficient time to get my work done

Appendix 2.

Table C. Full prediction model of 10-year risk for work disability

Predictor	b	(SE)	p-value
**Self-rated health**	**-0.318**	**0.012**	**<0.0001**
Jenkins sleep scale Q1	-0.0233	0.0075	0.0018
**Jenkins sleep scale Q2**	**-0.0229**	**0.0073**	**0.0017**
Jenkins sleep scale Q3	-0.0024	0.008	0.7618
Jenkins sleep scale Q4	-0.0097	0.0075	0.1978
**BMI**	**-0.1045**	**0.0874**	**0.2317**
**Smoking**	**-0.075**	**0.0199**	**0.0002**
Alcohol consumption	-0.1614	0.0243	<0.0001
Inactivity	-0.1572	0.0208	<0.0001
GHD	0.0039	0.0016	0.0185
Relational justice	-0.0416	0.0196	0.0332
Procedural justice	0.0341	0.0215	0.1124
Participatory safety	0.0044	0.0111	0.6913
Support for innovation	0.0033	0.0121	0.7882
Vision	-0.0246	0.0138	0.0751
Task orientation	-0.0179	0.0136	0.1871
**Sex**	**-0.025**	**0.0166**	**0.131**
**Age**	**0.0012**	**0.0163**	**0.9411**
**Socioeconomic position**	**-0.0598**	**0.0249**	**0.0163**
**No. of sickness absences during previous year**	**-0.3246**	**0.0076**	**<0.0001**
**Chronic illness**	**-0.1018**	**0.0054**	**<0.0001**
Job strain	-0.2917	0.0122	<0.0001
Effort-reward imbalance	-0.1685	0.0111	<0.0001
Shift work	-0.0756	0.0213	0.0004
Night shift	0.045	0.0296	0.129

Intercept = 6.7996
Scale = 0.6275073
Variables included in the final model are in bold.

Table D. Final prediction model of 10-year risk for work disability

Predictor	b (SE)	p-value
Age=35-39	-0.2339	<0.01
Age=40-44	-0.4356	<0.01
Age=45-49	-0.8825	<0.01
Age=50-54	-1.2873	<0.01
Age=55+	-1.5418	<0.01
BMI<18.5	-0.1724	0.14
BMI=25-30	-0.0668	<0.01
BMI=30+	-0.1753	<0.01
SEP=2	-0.0457	0.9
SEP=3	-0.3171	<0.01
SEP=4	-0.3213	<0.01
SEP=5	-0.546	<0.01
SEP=6	-0.5294	<0.01
SEP=7	-0.6597	<0.01
Smoking=YES	-0.1638	<0.01
Chronic illness=1	-0.2252	<0.01
Chronic illness=2	-0.4462	<0.01
Chronic illness=3	-0.5342	<0.01
Self-rated health=2	-0.2348	<0.01
Self-rated health=3	-0.5539	<0.01
Self-rated health=4	-1.1336	<0.01
Self-rated health=5	-1.5182	<0.01
Difficulty falling asleep=2	-0.0281	<0.01
Difficulty falling asleep =3	-0.0769	<0.01
Difficulty falling asleep =4	-0.1267	<0.01
Difficulty falling asleep =5	-0.2014	<0.01
Difficulty falling asleep =6	-0.2245	<0.01
No. Sickness absences in previous year=1	-0.4334	<0.01
No. Sickness absences in previous year=2	-0.7413	<0.01
No. Sickness absences in previous year=3	-1.133	<0.01

Intercept = 5.7912
Scale = 1.2046

Formula for calculating absolute risk for work disability in 10 years (x) is as follows.

$P(x) = \Phi[(\ln(10)\text{-linear prediction})/\text{scale})]$,
where Φ is the standard cumulative normal distribution

For an individual with following risk factors (age = 52, BMI= 23, SEP= 6, Smoking = no, chronic illness = 1, self-rated health =4, sleep = 3, sickness absences =1):
$P(x) = \Phi\ ((\ln(10)-(5.7912-1.2873-0.5294-0.2252-1.1336-0.0769-0.4334))/1.2046) = 0.434$

Appendix 3.

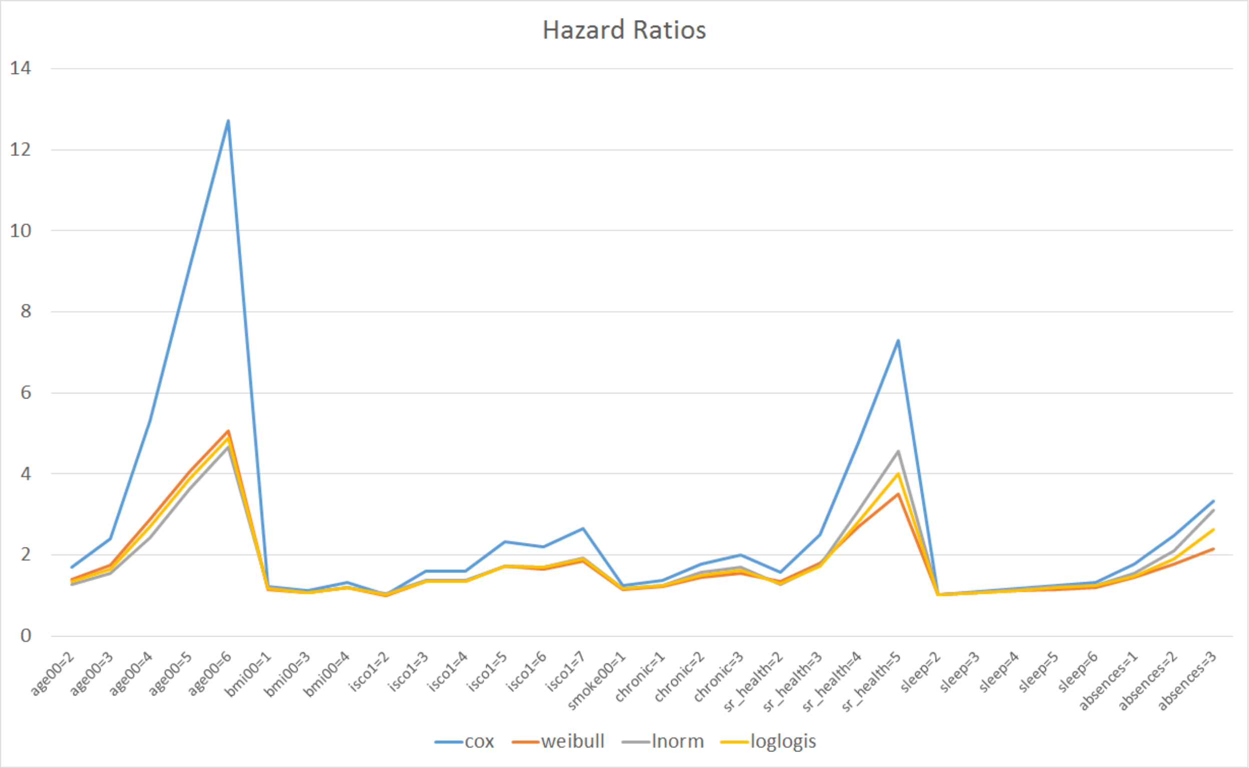

Figure A. Hazard ratios from Cox regression model and the three (Weibull, log-normal (lnorm), and log-logistic (loglogis)) best parametric survival models for the final prediction model.

Appendix 4.

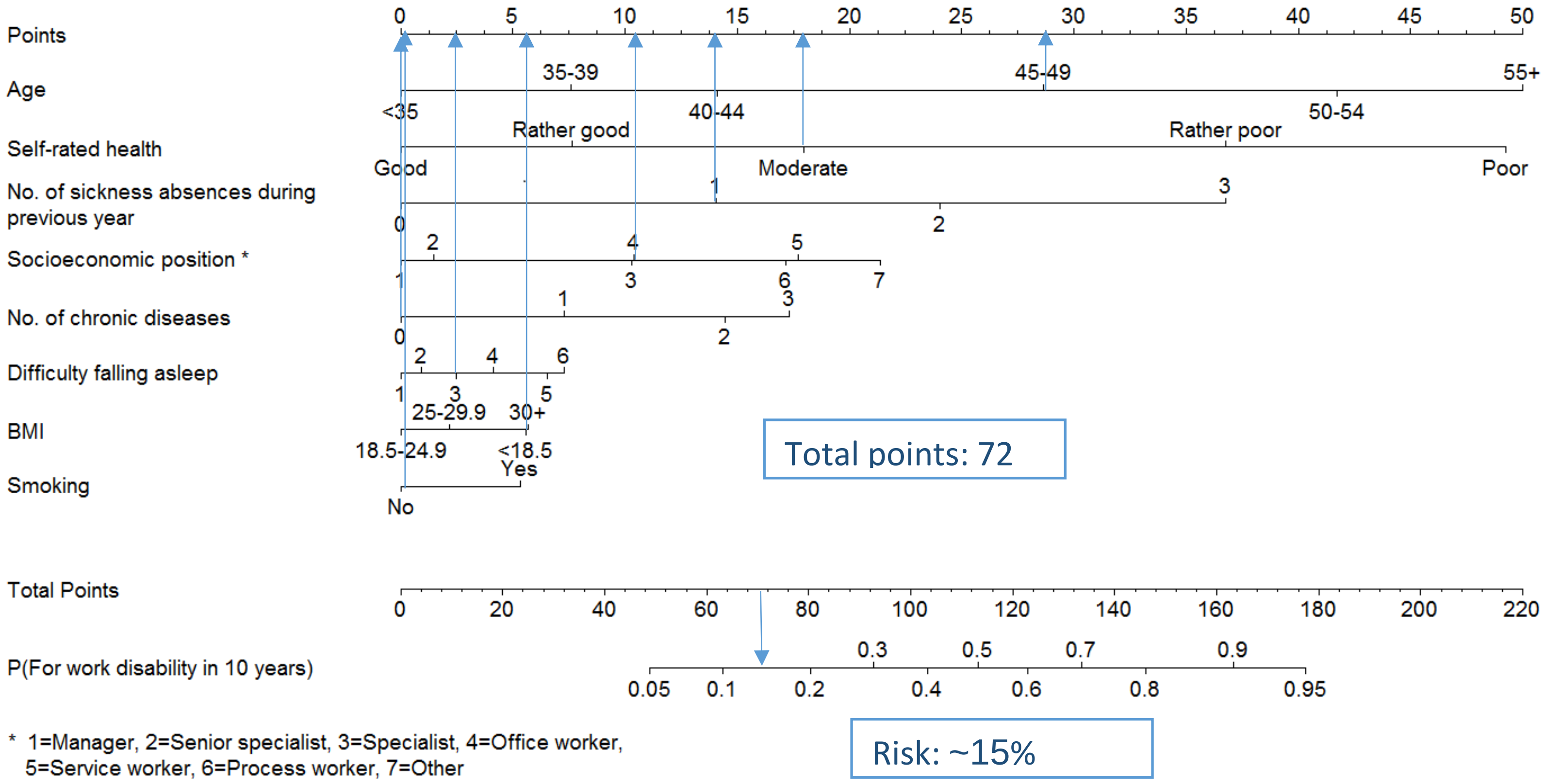

Figure B. Nomogram for the final model. For each predictor, find the appropriate value on the scale and read the corresponding points assigned to that value for the top most "Points" scale. Do this to all predictors and sum those points together. Then find that sum on the "Total points" scale and read the risk for work disability from the scale below. An example for 47 year old person with some risk factors is given in the figure (colored in blue).

Appendix 5.

Table E. Alternative prediction model with job strain scale

Predictor	b	p-value
Age=35-39	-0.3701	<0.001
Age=40-44	-0.6579	<0.001
Age=45-49	-1.2089	<0.001
Age=50-54	-1.7159	<0.001
Age=55+	-2.0489	<0.001
SEP=2	-0.1256	0.111
SEP=3	-0.4271	<0.001
SEP=4	-0.5408	<0.001
SEP=5	-0.8032	<0.001
SEP=6	-0.8796	<0.001
SEP=7	-0.9742	<0.001
Job strain =1	-0.2860	<0.001

Intercept = 5.8689
Scale = 1.3713

Formula for calculating absolute risk for work disability in 10 years (x) is as follows.
$P(x) = \Phi[(\ln(10)\text{-linear prediction})/\text{scale})]$,
where Φ is the standard cumulative normal distribution.

Appendix 6.

Table F. Alternative prediction model (2 work items)

Predictor	b	p-value
Age=35-39	-0.3685	<0.001
Age=40-44	-0.652	<0.001
Age=45-49	-1.1995	<0.001
Age=50-54	-1.7	<0.001
Age=55+	-2.0328	<0.001
SEP=2	-0.1389	0.0777
SEP=3	-0.4383	<0.001
SEP=4	-0.5424	<0.001
SEP=5	-0.7997	<0.001
SEP=6	-0.8859	<0.001
SEP=7	-0.9413	<0.001
Excessive amount of work =2	0.0065	0.8673
Excessive amount of work =3	-0.1051	0.0053
Excessive amount of work =4	-0.2566	<0.001
Excessive amount of work =5	-0.4386	<0.001
Repetitive work =2	0.0737	0.2548
Repetitive work =3	0.0358	0.5828
Repetitive work =4	-0.1002	0.0987
Repetitive work =5	-0.205	0.001

Intercept = 6.0169
Scale = 1.3647

Formula for calculating absolute risk for work disability in 10 years (x) is as follows.
P(x) = Φ[(ln(10)-linear prediction)/scale)],
where Φ is the standard cumulative normal distribution.

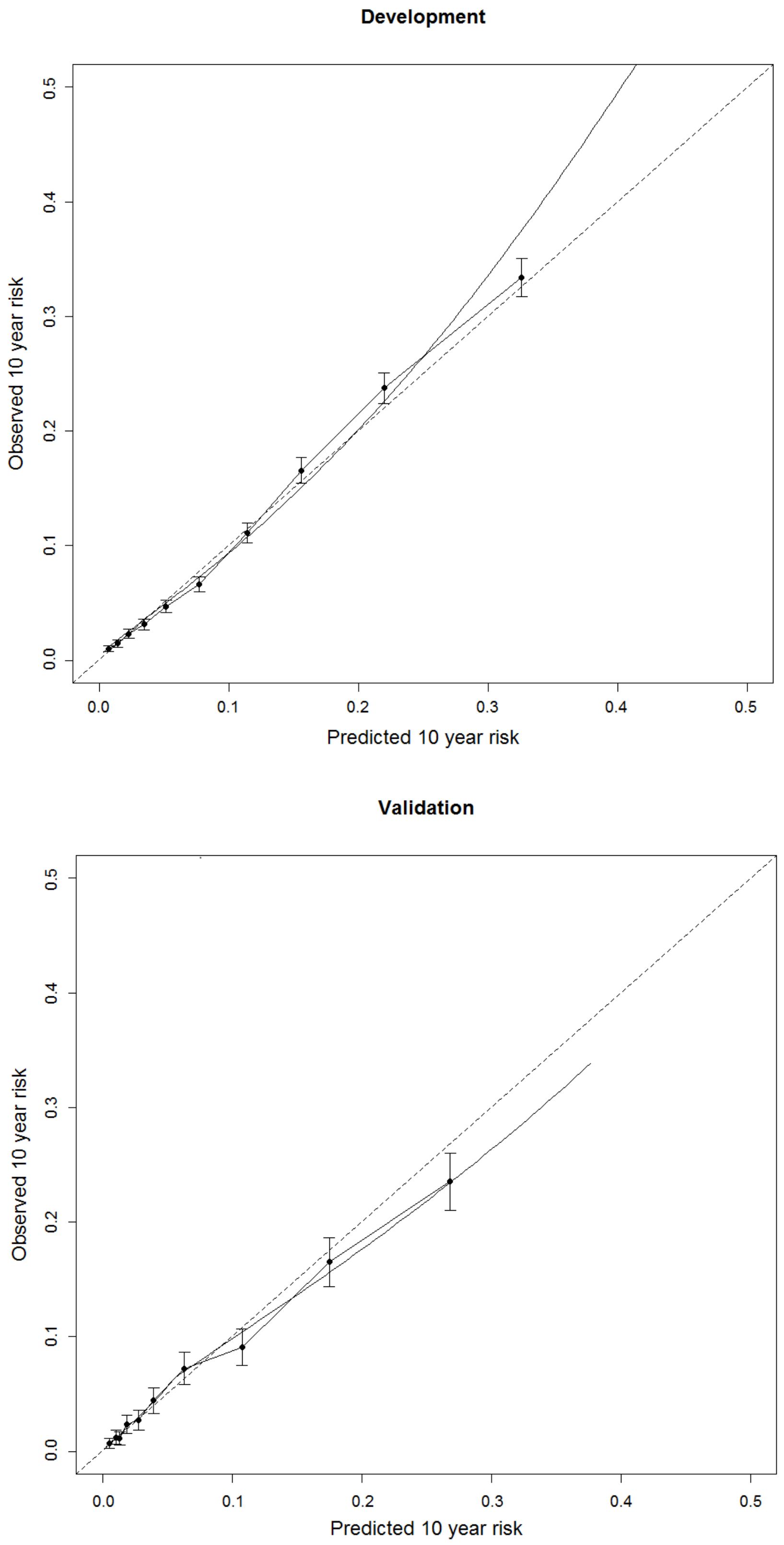

Figure C. Calibration plots for the alternative prediction model (2 work-related items).
